# Intensive feeding alters the rumen microbiota and its fermentation parameters in natural grazing yaks

**DOI:** 10.3389/fvets.2024.1365300

**Published:** 2024-04-05

**Authors:** Shichun He, Zaimei Yuan, Sifan Dai, Zibei Wang, Shusheng Zhao, Rongjiao Wang, Qing Li, Huaming Mao, Dongwang Wu

**Affiliations:** ^1^Key Laboratory of Animal Nutrition and Feed Science of Yunnan Province, Yunnan Agricultural University, Kunming, China; ^2^Kunming Animal Disease Prevention and Control Center, Kunming, China; ^3^Panzhihua Academy of Agricultural and Forestry Sciences, Panzhihua, China

**Keywords:** yak, intensive feeding, natural grazing, rumen fermentation, rumen microorganism

## Abstract

**Introduction:**

Amidst the challenging environmental conditions characterized by low oxygen levels and cold temperatures on the plateau, alterations in nutrient supply emerge as pivotal factors influencing the survival and reproduction of yaks. Intensive feeding stands out as a substantial mechanism for nutrient provision, initiating discernible changes in the host’s rumen flora. Within the extreme natural conditions prevailing in the plateau area of northwest Yunnan, China, there exists a con-strained comprehension of the variations in rumen microflora, fermentation parameters, and growth responses exhibited by yaks subjected to intensive feeding.

**Methods:**

This study employs 16S rRNA and ITS sequencing methods to scrutinize the rumen flora of yaks engaged in both natural grazing (G) and intensive feeding (F) on the plateau.

**Results:**

The outcomes unveil that, during the severe winter season, yaks adeptly modulate the abundance and diversity of rumen flora in response to dietary modifications under intensive feeding, aiming to optimize the efficient utilization of dietary fiber and energy. Principal Coordinate Analysis (PCoA) illustrates a substantial alteration in the rumen microbial community of naturally grazing yaks when exposed to intensive feeding. The natural grazing group manifests a higher prevalence of Firmicutes and Bacteroidetes, while the intensive feeding group exhibits heightened levels of *Prevotella* in the rumen. The *Rikenellaceae* _ RC9 _ gut_ group, associated with mycobacteria, prevails more abundantly in the natural grazing setting. PICRUSt2 analysis indicates that intensive feeding induces bacterial gene overexpression linked to protein metabolism. Rumen fungi showcase heightened diversity under intensification. Intensive feeding results in an augmented abundance of non-fiber-degrading bacteria and semi-fiber-degrading bacteria, accompanied by elevated concentrations of Volatile Fatty Acids (VFA).

**Discussion:**

These findings yield novel insights into the shifts in the rumen microflora of yaks acclimated to intensive feeding in high-altitude environments, provide an important reference for the nutritional regulation of supplemental feeding of natural grazing yaks in the cold season, ultimately contributing to their enhanced growth.

## 1 Introduction

The elevated plateau of the Diqing Tibetan Autonomous Prefecture in the northwest region of Yunnan Province, China, stands as a crucial pastoral zone. Distinguished by its high altitude, diminished oxygen levels, and intense ultraviolet radiation, the locale presents a formidable challenge to the survival of mammals (1–4). Among the vital livestock breeds in this region are yaks (*Bos grunniens*), whose body shape, appearance, and digestive system have undergone evolutionary adaptations through a protracted period of natural selection and deliberate domestication (5–7). These adaptations equip yaks to contend with harsh environmental factors, including high altitudes, hypoxia, strong ultraviolet radiation, and nutrient deficiencies (8–10). Notably, the intestinal microbiota within the digestive system of yaks has coevolved with the host, yet remains dynamically influenced by alterations in diet and environmental conditions (11, 12). Mao et al. (13) conducted a 16S rRNA study on the rumen of Holstein cows and found that the relative abundance of Lentisphaerae increased with increasing dietary protein and energy levels in the diet and the study showed that the abundance of Len-tisphaerae was significantly influenced by isobutyric, valeric and isovaleric acids. Petri et al. (14) showed that changes in diet structure affected the relative abundance of Prevotella in the rumen of yellow cattle and that the proportion of Prevotella increased significantly with increasing levels of nitrogenous compounds in the diet. These microbial communities play a pivotal role in nutrient assimilation, enhancement of immune functions, and the overall growth and development of the host. The rumen, in particular, harbors the richest and most diverse microbial community in yaks (15). The elevated pastures of the plateau exhibit elevated levels of intricate plant polysaccharides, necessitating the role of rumen microorganisms in providing enzymes to convert these polysaccharides into volatile fatty acids (16). These microorganisms, in turn, are effectively utilized by the host. Although an increasing number of studies have attempted to elucidate the flora composition of yak rumen microbes, it has been found that the stability of rumen microbes is higher in grazing yaks compared to housed yaks, especially in winter when pasture quality is poor, reflecting the higher feeding and utilization efficiency of yaks on low and rough winter pasture (17, 18). Currently, there is a paucity of studies addressing the alterations in rumen flora resulting from natural host grazing induced by the intensive feeding of ruminants in plateau areas. An imperative undertaking is to delve into the impacts of intensive feeding on the interplay between dietary nutrition and the rumen flora of naturally grazing yaks in the western Yunnan plateau. This research holds particular significance in elucidating how the yak rumen flora adapts to the plateau environment and, consequently, sheds light on how intensive feeding influences the survival and health of mammals amidst extreme environmental conditions.

To explore the interaction between intensive feeding and rumen microbiota, we performed comprehensive analyses, including 16S rRNA and ITS gene analysis, as well as volatile fatty acid analysis, to assess the impact of intensive feeding on the host rumen microbiota. These thorough investigations contribute valuable insights into under-standing the adaptability of yaks to the challenging high-altitude environment. Provide an important reference for the nutritional regulation of supplemental feeding of natural grazing yaks in the cold season.

## 2 Materials and methods

### 2.1 Animals, diets, and experimental design

The experiment was conducted in the pasture of Tiancheng Lun Zhu Agricultural Products Development Co., Ltd., from August to November 2022. Male yaks that were 3 years old, with similar body weight, and in good condition were selected and randomly divided into 2 groups: natural grazing group (G, *n* = 9) and intensive feeding group (F, *n* = 10). Yaks in the G group grazed freely in the pasture. In the F group, the yaks were fed whole corn silage, and 3 kg of reserve concentrate supplement was fed to each head. The nutritional composition of the G group and F group are shown in Table 1. This experimental period was 100 d, including 10 d of pretesting and 90 d of main testing. Rumen fluid samples were collect-ed at the end of the test before morning feeding.

**Table 1:** The nutritional composition (% DM basis) of different diets.

Group	DM	CP	EE	Ash	Ca	P	NDF	ADF	ADL
G	46.50	8.52	2.50	6.12	0.85	0.29	51.52	34.99	6.63
F	44.85	10.48	1.68	10.97	0.61	0.65	53.21	26.81	2.98

### 2.2 Sample collection and measurements

At the end of this experiment, rumen fluid was collected using a bendable oral gastric tube with a metal filter before feeding in the morning. Discard about 50 mL of newly collected samples to avoid saliva contamination. About 300 mL of rumen fluid was then extracted, part of which was used for pH determination and the rest were divided into 15 mL sterile centrifuge tubes and stored in liquid nitrogen for the determination of ruminal fermentation parameters and microbiota analysis. Oral tube washed before taking sample from next animal.

Measuring rumen pH with a pH meter (PHS-10 portable pH meter), and the pH meter was calibrated with the appropriate calibration solution before measurement. NH_3_-N was determined by the phenol-hypochlorous acid colorimetric method. 40 μL of supernatant was taken into a labelled test tube after rumen fluid centrifugation at 12000 × g for 20 min, 2.5 mL of phenol chromogenic agent and 2.0 mL of sodium hypochlorite reagent were added. After that, the supernatant was thoroughly mixed by shaking and placed in a 37°C water bath for 30 min. Colorimetric analysis of the supernatant was performed using a visible spectrophotometer at a wavelength of 550 nm. Repeat the above steps using NH_4_Cl standard solution instead of rumen fluid and plot the standard curve. The NH_3_-N concentration was calculated from the regression equation, colorimetric results and standard curve.

Volatile fatty acids (VFA) were determined by gas chromatography–mass spectrometry. Take the sample into the 1.5 mL EP tubes. Add 0.05 mL 50% H_2_SO4 and 0.2 mL of extracting solution (25 mg/L stock in methyl tert-butyl ether) as internal standard, vortex mixing for 30 s, oscillations in 10 min, then ultrasound treated for 10 min (incubated in ice water). Centrifuge for 15 min at 10000 rpm, 4°C. Keep at −20°C for 30 min. The supernatant was transferred to fresh 2 mL glass vial for GC–MS analysis. SHIMADZU GC2030-QP2020 NX gas chromatography mass spectrometer is used. The system utilized an HP-FFAP capillary column. 1 μL aliquot of the analyte was injected in split mode (5: 1). Helium was used as the carrier gas, the front inlet purge flow was 3 mL min^−1^, and the gas flow rate through the column was 1 mL min^−1^. The initial temperature was kept at 50°C for 1 min, then raised to 150°C at a rate of 50°C min^−1^ for 1 min, then raised to 170°C at a rate of 10°C min^−1^ for 0 min, then raised to 210°C at a rate of 20°C min^−1^ for 1 min, then raised to 240°C at a rate of 40°C min^−1^ for 1 min. The injection, transfer line, quad, and ion source temperatures were 220°C, 240°C, 150°C and 200°C. The energy was-70 eV in electron impact mode. The mass spectrometry data were acquired in Scan/SIM mode with the m/z range of 33–150 after a solvent de-lay of 3.0 min.

### 2.3 DNA extraction and sequencing

The microbial community DNA was extracted using the EZNA Stool DNA Kit (TianGen, China, Catalog: DP712) following the manufacturer’s instructions. 16S rRNA/ITS genes of distinct regions were amplified using a specific primer with the barcode. All PCR reactions were carried out with 15 μL of Phusion^®^ High-Fidelity PCR Master Mix (New England Biolabs); 2 μM of forward and reverse primers, and about 10 ng template DNA. Thermal cycling consisted of initial denaturation at 98°C for 1 min, followed by 30 cycles of denaturation at 98°C for 10 s, annealing at 50°C for 30 s, and elongation at 72°C for 30 s and 72°C for 5 min. Mix the same volume of 1X loading buffer (containing SYB green) with PCR products and operate electrophoresis on 2% agarose gel for detection. PCR products were mixed in equidensity ratios. Then, the mixture of PCR products was purified with a Universal DNA Purification Kit (TianGen, China, Catalog: DP214). Sequencing libraries were generated using NEB Next^®^ Ultra™ II FS DNA PCR-free Library. Prep Kit (New England Biolabs, United States, Catalog: E7430L) following the manufacturer’s recommendations and indexes were added. The library was checked with Qubit and real-time PCR for quantification and bioanalyzer for size distribution detection. Quantified libraries were pooled and sequenced on Illumina platforms, according to effective library concentration and data amount required.

### 2.4 Statistical analysis

The rumen fluid samples were sent to Yunnan Pulis Biotechnology Co., Ltd. for sequencing, and 16S rRNA and ITS technologies were used to determine the diversity of the rumen microflora. DNA was extracted from the rumen fluid samples and the quality of the DNA was assessed by 1.0% agarose gel electrophoresis. The raw data of each sample was first obtained by splitting according to the barcode, and the barcode and primers were removed, followed by splicing of R1 and R2 sequence data using FLASH software. The spliced tags were subjected to quality control to obtain clean tags, and then chimera filtering was performed to obtain effective data that could be used for subsequent analyses. Each deemphasized sequence ASV (Amplicon Sequence Variants) (19) generated after noise reduction using DADA2 (20) was used to annotate species using the QIIME2 classifysklearn (21) algorithm for each ASV using a pretrained Naive Bayes classifier. Based on the results of the ASV annotation and the feature list of each sample, the results of species classification, diversity index, and community nodes were obtained. To analyze the diversity, richness, and uniformity of the communities in the sample, alpha diversity in QIIME2, alpha diversity was calculated Observed_otus, Chao1and Shannon. In the analysis of inter-group difference of Alpha diversity index, *T*-test and wilcox rank sum test were used to analyze whether there were significant differences in species diversity between groups. We perform PCoA analysis based on Weighted_unifrac and Unweighted_unifrac, and select the principal coordinate combination with the largest contribution rate for presentation. The original 16S rRNA/ITS data were available in the NCBI SRA database with accession numbers PRJNA10366001, and PRJNA1034800.

Independent samples *t*-test based on SPSS was applied to compare rumen fermentation parameters between the natural grazing group and intensive feeding group, and the differences were considered statistically significant at *p* < 0.05.

## 3 Results

### 3.1 Rumen fermentation parameters

The effect of the G group and F group on rumen fermentation parameters in yaks is shown in Table 2. The NH_3_-N, TVFA (Total volatile fatty acids), isobutyric acid, isovaleric acid, pentanoic acid and caproic acid of the F is significantly higher than that of the G group (*p* < 0.05).

**Table 2:** The effects of the natural grazing group and intensive feeding group on the rumen fermentation parameters of the yaks.

Item	G	F	*p* value
pH	6.97 ± 0.20	6.77 ± 0.39	0.16
NH_3_-N(mg/dl)	6.16 ± 2.14^b^	16.29 ± 7.06^a^	<0.01
Total volatile fatty acids (mmol/L)	17.23 ± 3.92	28.80 ± 10.59	0.01
Acetic acid (mmol/L)	10.21 ± 4.45	14.71 ± 4.56	0.12
Propionic acid (mmol/L)	7.13 ± 1.15	7.80 ± 3.73	0.56
Isobutyric acid (mmol/L)	0.46 ± 0.14	1.03 ± 0.31	<0.01
Butyric acid (mmol/L)	4.57 ± 1.21	3.66 ± 1.64	0.19
Isovaleric acid (mmol/L)	0.24 ± 0.10	0.86 ± 0.30	<0.01
Pentanoic acid (mmol/L)	0.27 ± 0.09	0.54 ± 0.26	0.01
Caproic acid (mmol/L)	0.03 ± 0.01	0.10 ± 0.07	0.01
Acetic acid/ Propionic acid	1.74 ± 0.87	2.18 ± 0.77	0.37

Natural grazing group (G), Intensive feeding group (F), the same below.

### 3.2 Rumen bacteria influenced by natural grazing group and intensive feeding group

**Figure 1:**
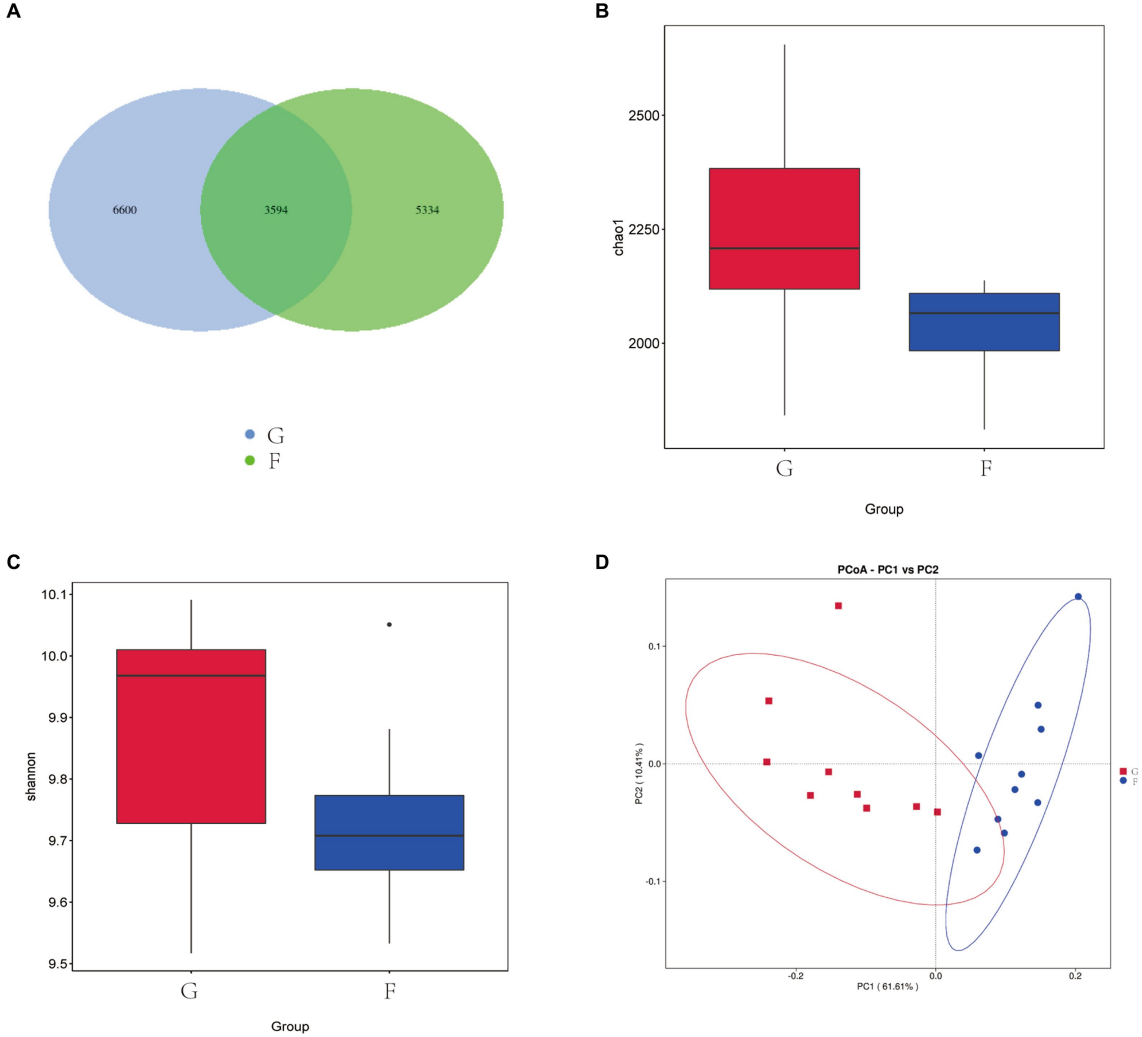
Effects of natural grazing group and intensive feeding group on rumen mi-crobial flora α-diversity and β-diversity of yaks. **(A)** OTU Venn diagram. **(B)** Chao index. **(C)** Shannon index. **(D)** PCoA. Natural grazing group (G), Intensive feeding group (F), same as below.

As shown in Figure 1A, A total of 15,528 OTU were identified in the two experimental groups. The G had 10,194 OTU, the F group had 8,928 OTU. A total of 3,594 OTU were present in the two experimental groups. The α diversity and β-diversity analysis showed that there were differences in the abundance of microflora between the two groups, and the Chao1 (Figure 1B) and Shannon index (Figure 1C) of the G group was significantly higher than that of the F group (*p* < 0.05). This indicates that intensive feeding increased the abundance of microflora in the rumen of yaks. According to PCoA (Figure 1D), significant differences were observed between the rumen bacteria of the G group and F group. PC1 and PC2 of the microflora community accounted for 61.61 and 10.41% of the variation among samples.

**Figure 2:**
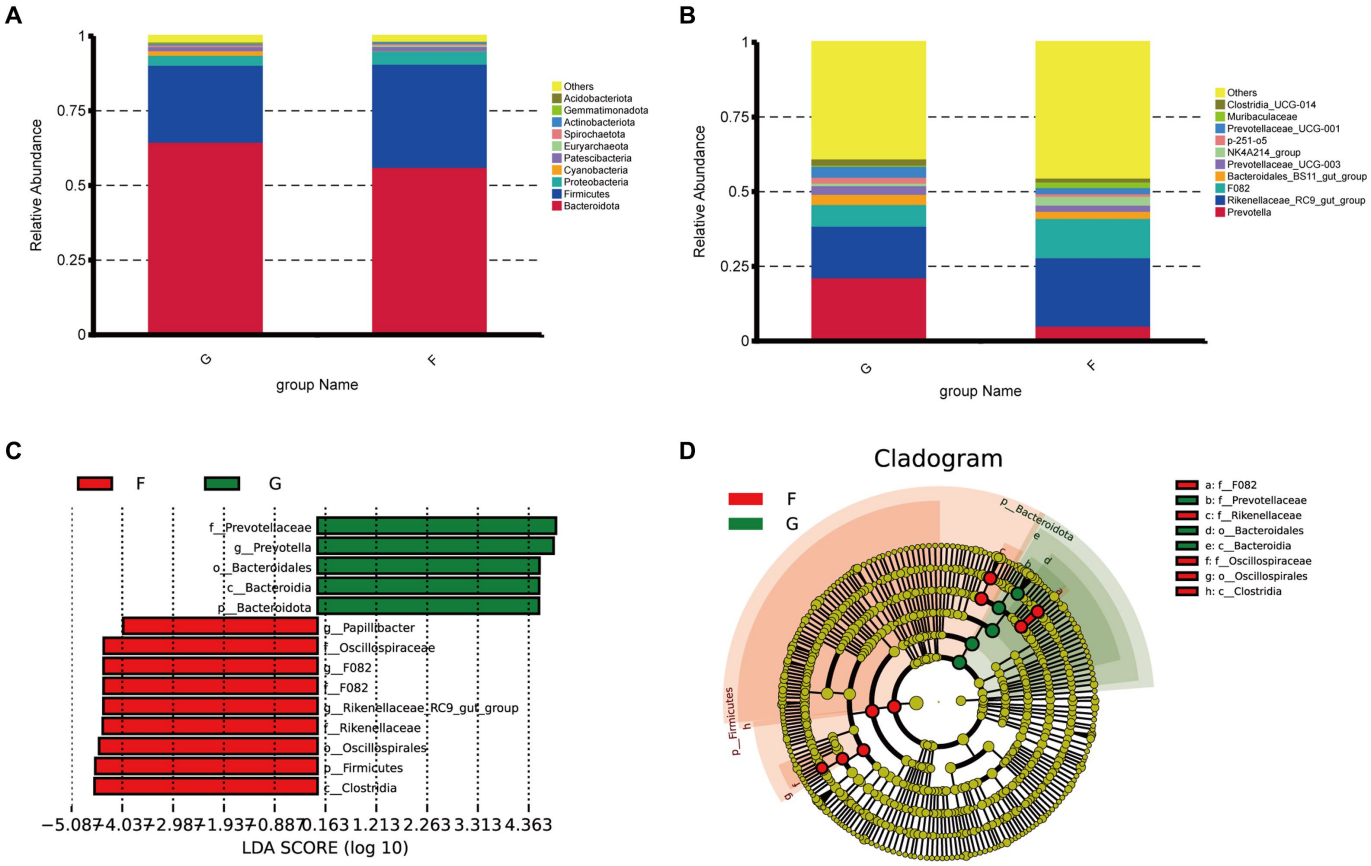
Classification of rumen microbial flora composition in natural grazing group and intensive feeding group of yaks. **(A)** Comparison of dominant phyla in the different groups. **(B)** Comparison of dominant genera in the different groups. **(C,D)** Cluster plots were analyzed by LEfSe. Different colors represent different groups, nodes of different colors represent the microbiota that play an important role in the group represented by the color, a color circle represents a biomarker, and the legend in the top right corner shows the name of the biomarker.

The microbial community of the rumen and the rumen formation parameters were influenced by the intake and diet. We performed a taxonomic analysis of the 10 bacterial phyla identified. The rumen microorganisms of yaks under the G group and F group were dominated by three dominant phyla at the phylum level, namely Firmicutes, Bacteroidetes, and Proteobacteria. The proportions of Firmicutes, Bacteroidetes, and Proteobacteria were 25.79 and 34.57%, 64.70 and 56.31%,33.32 and 4.36% in the G group and F group (Figure 2A).

It can be seen from Figure 2B, that dominant yak rumen microorganisms at the genus level under the natural grazing group and intensive feeding groups were *Prevotella* and *Rikenellaceae*_RC9_gut_group at the genus level, *Prevotella* was 21.42 and 5.27%. The *Rikenellaceae_*RC9_gut_group belonged to the phylum Mycobacterium with 17.27 and 22.89% in the G group and F group, and the G group was significantly higher than the F group. The next most dominant genera were *F082*, *Ruminalococcaceae* (NK4A214_group), *Bacteroi-dales_BS11_gut_group*, *Prevotellaceae_*UCG-001.

Distribution map of LDA value and evolutionary branch map of different species of rumen bacteria in yaks under natural grazing and intensive feeding. LEfSe analysis was performed to identify the bacteria in G group and F group (Figure 2C). A total of 8 bacteria were listed as signature microbiota for the two groups. The signature rumen microbiota in the G group included Prevotellaceae, Bacteroidsles, and Bacteroides. Oscillqapiraceae, *F082*, Rikenellaceae, Os-cillospirales, and Clostridia in the F group.

**Figure 3:**
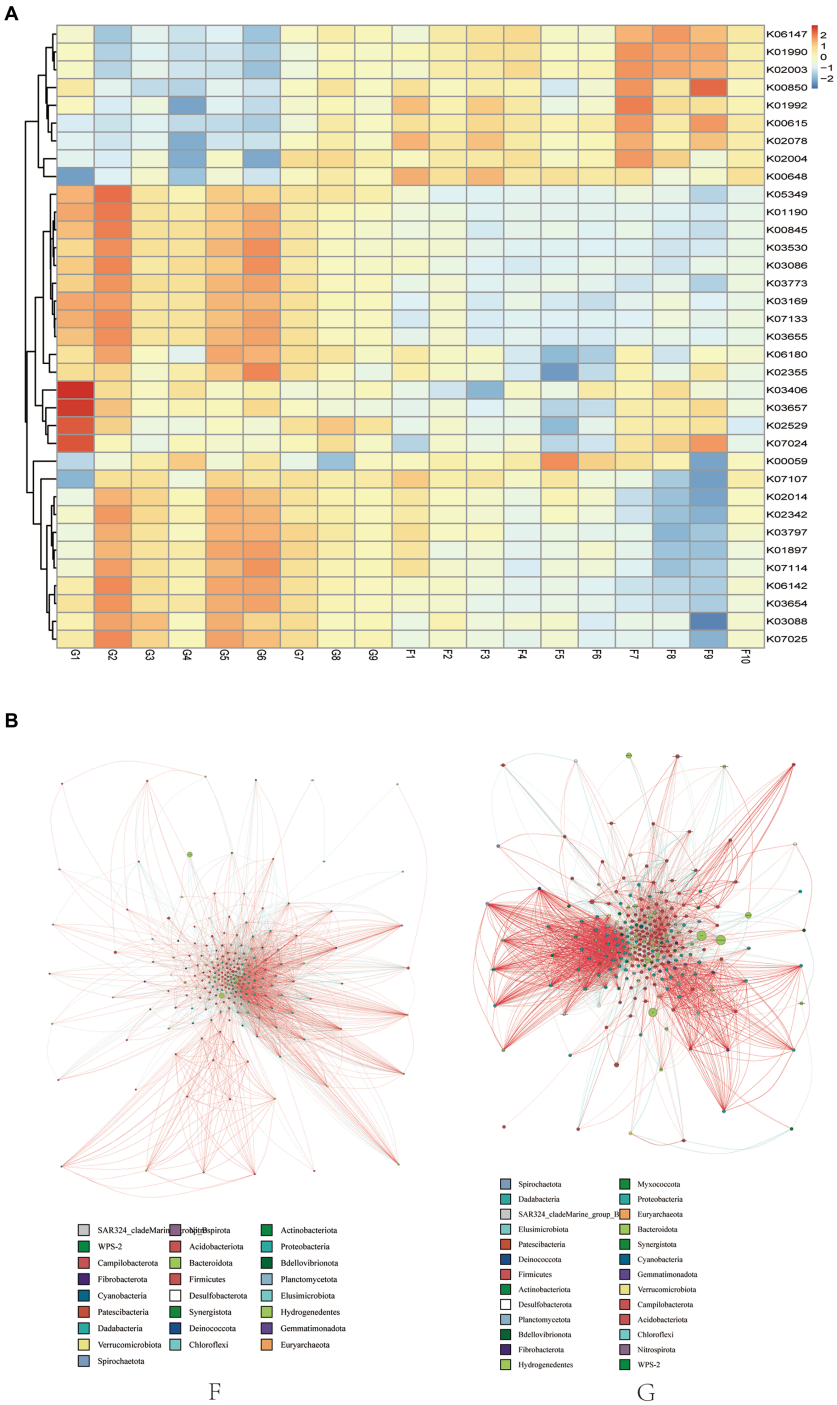
Microbial interactions between rumen bacterial communities in yak. **(A)** Heat map of functional predicted abundance. According to the functional annotations and abundance information of the samples in the database, the top 35 functions with the highest abundance and their abundance information in each sample were selected to draw heat maps, and the clustering was carried out from different functional levels. Show the results of the functional prediction analysis of the KO database. **(B)** Network diagram. Different nodes represent different genera, node size represents the average relative abundance of the genus, nodes of the same phylum have the same color (as indicated in the figure legend), the absolute value of the correlation coefficient between line thickness and species interactions between nodes is positive, and the correlation coefficient between line color and correlation is positive (red is positively, blue is negatively).

Significant differences in functional abundance between groups G and F were observed when predicted yak rumen microbiome functions using PICRUSt (Figure 3A). The relative abundance of dominant genes (K01990), (K01992), (K02004), (K02003), (K00648), (K02004), (K02078), and (K06147) increased in the F group, most of these genes are associated with protein metabolism. The relative abundance of dominant genes (K01190), (K00845), (K02529), and (K07024) increased in the G group, these genes are associated with energy metabolism. Regarding enzymes, bacteria associated with the outer membrane receptor protein of the iron complex (K02014) were more abundant in G Group.

Microbial networks were used to analyzed microbial interactions between rumen bacterial communities in yaks. The results showed that the positive correlation of competition between different species of the same genus was observed by constructing the cooccurrence network diagram of microorganisms of the same genus, and the positive correlation between species was more frequent than the negative correlation (Figure 3B). These phenomena indicated that the competition between species in the natural grazing genus was lower than that in the intensive feeding group, and thus the rumen microecological community structure was more closely related.

### 3.3 Rumen fungi influenced by natural grazing group and intensive feeding group

**Figure 4:**
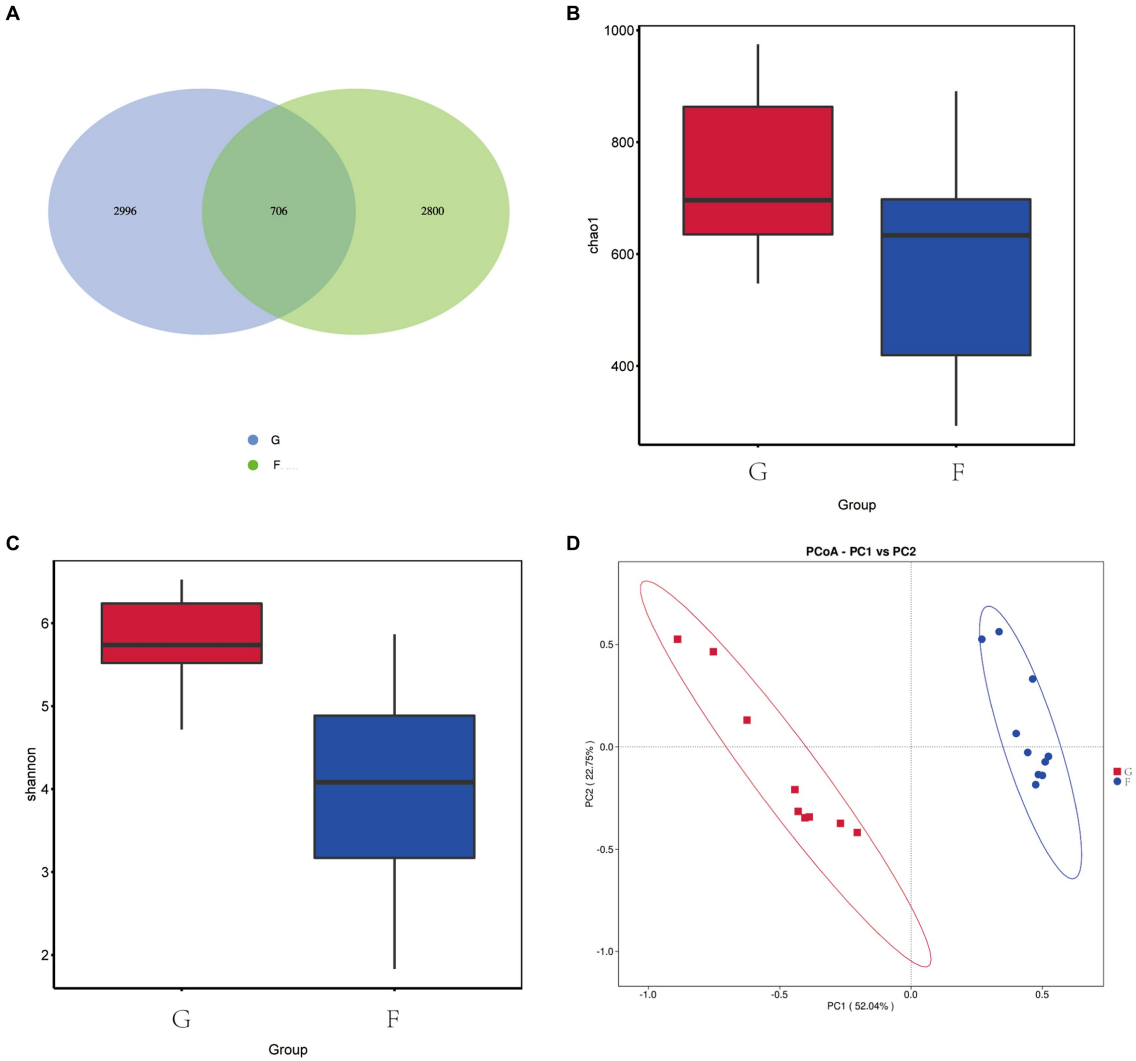
Effects of natural grazing group and intensive feeding group on rumen fungi α diversity and β-diversity of yaks. **(A)** OTU Venn diagram. **(B)** Chao index. **(C)** Shannon index. **(D)** PCoA.

As shown in Figure 4A, a total of 6,212 OTU was identified in the G group and F group. The G group had 3,702 OTU, the F group had 3,506 OTU, and a total of 706 OTU were present in the two experimental groups. The α diversity and β-diversity analysis showed that there were differences in the abundance of microflora between the two groups. The richness of the fungal communities was indicated by the Chao1 index, with higher values indicating greater richness. The Chao1 index (Figure 4B) in the F group was higher than that in the G group. The Shannon index is a composite measure of community richness and evenness. Higher values indicate greater diversity and a more even distribution of fungi species. The F group had significantly higher Shannon index (Figure 4C) compared to the G group. According to PCoA (Figure 4D), significant differences were observed between the rumen fungi of the G group and F group. PC1 and PC2 of the fungi community accounted for 52.04 and 22.75% of the variation between groups.

**Figure 5:**
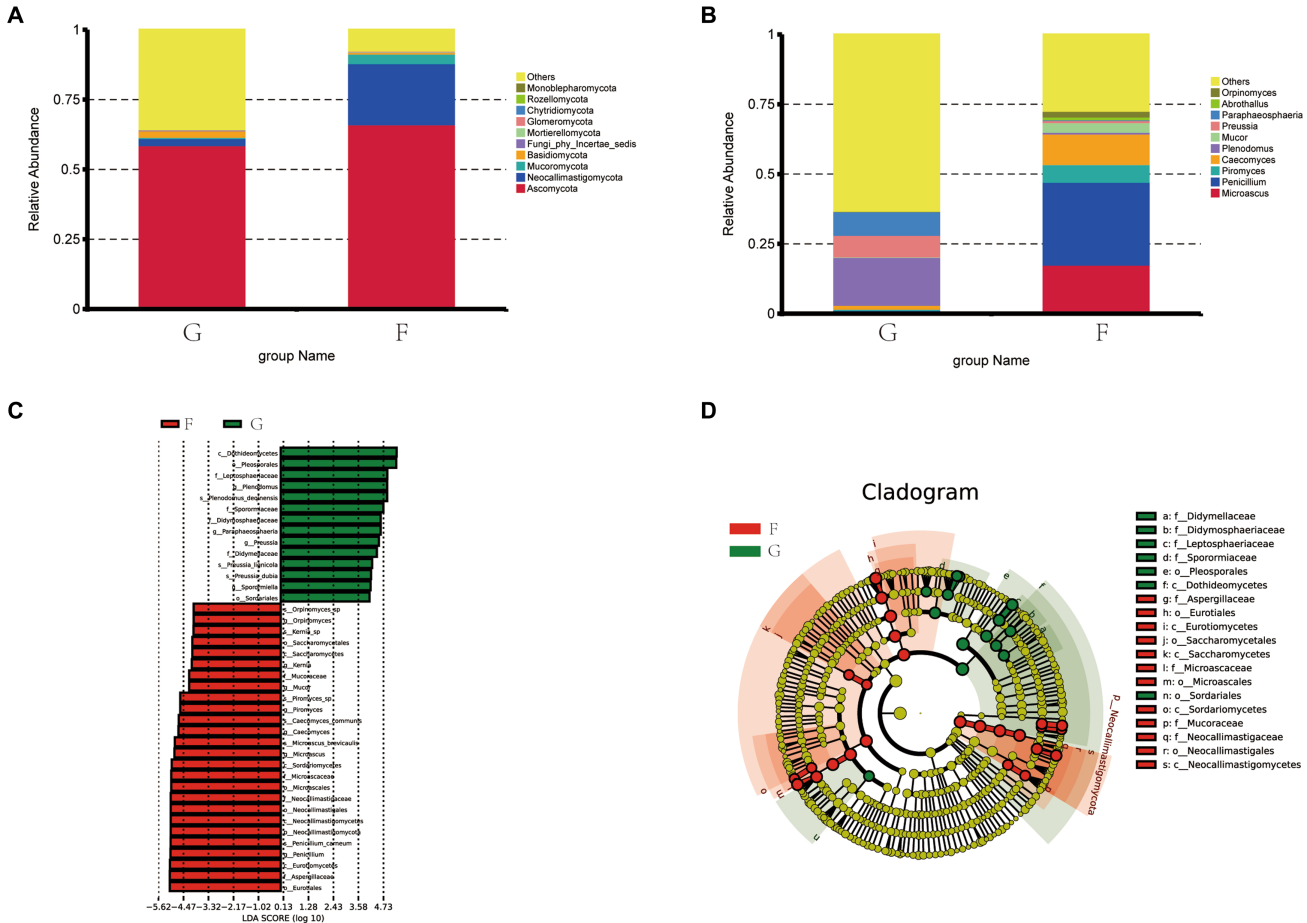
The composition of fungi in the rumen fluid of yak under the natural grazing group and intensive feeding group. **(A)** Comparison of dominant phyla in the F group and G group. **(B)** Comparison of dominant genera in the F group and G group. **(C,D)** Cluster plots were analyzed by LEfSe.

Figure 5 shows that Ascomycota and Neocallimastigomycota are the dominant fungi phyla in yak rumen fluid. The proportions of Ascomycota and Neocallimastigomycota were 58.88 and 66.17%, 2.58, and 21.90% in the G group and F group. The next most dominant phyla were Mucoromycota and Basidio-mycota, the proportions of Mucoromycota and Basidiomycota were 0.44 and 3.44%, 2.34 and 0.60% in the G group and F group.

Figure 5B shows the top ten fungi genera with their relative abundance. *Penicillium*, *Microascus*, *Caecomyces*, and *Piromyces* were the dominant genera in the F group and their abundance was 29.64, 17.61, 10.92%, and 3.67. *Plenodomus*, *Paraphaeosphaeria*, *Preussia*, and *Caecomyces* were the dominant genera in the G group and their abundances were 17.14, 8.17, 7.75, and 1.39%.

Distribution map of LDA value and evolutionary branch map of different species of rumen fungi in yaks under G group and F group. LEfSe analysis was performed to identify the fungi’s different feed methods (Figure 5C). A total of 19 fungi were listed as signature microbiota for the two groups. The signature rumen fungi in the G group included Dothideomycetes, Pleosporales, Leptosphaeriaceae, Sporormiaceae, Didymosphaeriaceae, Didymellaceae, and Sordar-iale. Aspergillaceae, Eurotiales, Eurotiomycetes, Saccharomycetales, Saccharomycetes, Microascales, Sordariomycetes, Mucoraceae, Neocallimastigaceae, Neocallimastigales, and Neocallimastigomycetes in the F group.

**Figure 6:**
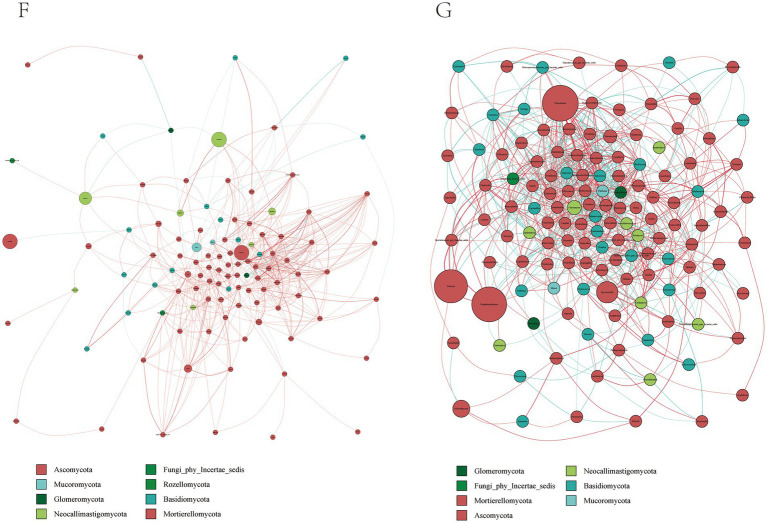
Microbial interactions between rumen fungi communities in yak. Different nodes represent different genera, node size represents the average relative abundance of the genus, nodes of the same phylum have the same color (as indicated in the figure legend), the absolute value of the correlation coefficient between line thickness and species interactions between nodes is positive, and the correlation coefficient between line color and correlation is positive (red is positively, blue is negatively).

By constructing the cooccurrence network diagram of fungi in the same genus, close competition correlation was found among different species in the same genus, and the positive correlation between species in the G group was higher than the negative correlation frequency (Figure 6), and the correlation between the F Group was not higher than that in the G group. These phenomena indicate that species in the G group were more competitive than those in the F group. Therefore, there is a great relationship between the community structure of rumen fungi.

## 4 Discussion

### 4.1 Effects of natural grazing group and intensive feeding group on the rumen fermentation parameters of yaks

A stable intra-rumen environment is particularly important for ruminants, rumen pH, NH_3_-N and VFA molar concentrations are important indicators of the stability intra-rumen environment, reflecting the state of rumen fermentation (22). The level of carbohydrates in the ration and the concentration of organic acids in the rumen determine the pH of the rumen fluid of ruminants, and the pH of the rumen fluid can effectively reflect the fermentation status of rumen microorganisms (23). Under the conditions of this experiment, the rumen pH of the intensive feeding group was lower than that of the natural grazing group, which may be attributed to the decrease in pH due to the increased intake of non-structural carbohydrates by yaks under the intensive feeding group, which were rapidly fermented in the rumen and produced large amounts of substances such as VFA. The NH_3_-N concentration in the intensive feeding group was significantly higher than that in the natural grazing group, which may be due to the high content of soluble proteins, easily digestible carbohydrates, and non-protein nitrogenous substances in the concentrate, and the slow rate of degradation, which favors the synthesis of NH_3_-N. This result is similar to the findings of Agle et al. (24) that an increase in roughage in the diet of dairy cows resulted in a decrease in rumen fluid NH_3_-N concentration, probably due to the structural carbohydrates in the roughage that reduced ammonia production.

Rapid rumen fermentation following ingestion of feed results in significant VFA production. The concentration and proportion of VFA in the rumen are primarily influenced by diet composition (25). When the diet is predominantly roughage, it contains high levels of cellulose and hemicellulose, with low levels of easily fermentable starch and soluble sugars. The carbohydrates are predominantly structural carbohydrates that are difficult to ferment, resulting in low production of volatile fatty acids. Under the current experimental conditions, rumen concentrations of TVFA, acetic acid, propionic acid, and butyric acid were significantly higher in the intensive feeding group than in the natural grazing group. This suggests that the diets were balanced under the housed conditions. The increase in rumen TVFA concentration resulted in the degradation of dietary cellulose, thereby improving nutrient digestibility and facilitating yak fattening.

### 4.2 Effects of natural grazing group and intensive feeding group on the rumen microbial bac-teria of yaks

The alpha diversity index is a comprehensive measure of the richness and diversity of the microflora. The higher the alpha diversity, the more complex and stable the composition of the flora, which increases its resistance and adaptability to external perturbations and benefits the health of the host (26). Liu et al. (2) discovered that increasing the concentration in the diet notably decreased the Chao1 index and Shannon index of yak rumen flora. The likely cause was the decreased pH of the rumen fluid, which resulted in the suppression of fiber-degrading bacteria growth and proliferation. In this experiment, the Chao1 and Shannon index of the G group were significantly higher than those of the F group. This suggests that the diversity of the rumen flora of yaks in the G group was significantly greater than that of the F group under experimental conditions.

The composition and nature of the feed shape the composition and variety of the rumen microflora. Many investigations in ruminants have demonstrated that the Firmicutes and Bacteroides constitute the prevailing phyla of rumen microflora in ruminants (27). The phyla with Firmicutes are mainly responsible for breaking down fibrous materials, while the Bacteroides are responsible for breaking down non-fibrous materials (28, 29), which plays a crucial role in the nutrient metabolism of ruminants. The most dominant phyla found in the microorganisms of yak rumen are Bacteroidetes, Firmicutes, and Proteobacteria (30). The proportions of Firmicutes, Bacteroidetes, and Proteobacteria were 25.79 and 34.57%, 64.70 and 56.31%, 4.36 and 3.32% in the G group and F group, respectively. The relative abundance of Firmicutes was significantly higher in the G group than in the F group. Proteobacteria were involved in the fermentation and digestion of soluble carbohydrates in the rumen of the ration (30), and the relative abundance of Proteobacteria in the F group was higher than that in the G group, indicating that the digestion and absorption efficiency was improved of yaks in the F group.

Many microorganisms are sensitive to dietary substrate concentration, and prolonged feeding of diets with different nutrient levels can cause changes in the number and structure of the rumen flora. Henderson et al. (31) studied rumen microbiota in 32 species, including buffalo, bison, sheep, goats, deer, alpaca, and greater alpaca, and found that dietary changes caused changes in the genus of microorganisms in the rumen. The main genera present in this experiment were *Prevotella* and *Rikenellaceae* _ RC9 _ gut_ group, both classified under Bacteroidetes. As one of the main protein-degrading bacteria in the rumen, *Prevotella* is involved in the breakdown of starch and protein. The increase in protein concentration will lead to a large number of *Prevotella*. *Rikenellaceae* _ RC9 _ gut_ group promotes lipid metabolism and is involved in the production of short-chain fatty acids, especially propionate (32). The *Rikenellaceae* _ RC9 _ gut_ group was also positively correlated with the growth performance of the host animals, and its abundance was higher in the animals with the best growth performance (33). Fernando et al. (34) demonstrated a greater proportion of Bacteroidetes relative to other rumen microorganisms in beef cows consuming concentrates over those fed green hay. In this experiment, the genus with the highest percentage of Bacteroidetes was *Rikenellaceae* _ RC9 _ gut_ group, which is consistent with Fernando’s findings and may be attributed to the higher crude protein and starch content of the concentrate supplements in the F group, which can effectively promote the proliferation of protein and starch degrading bacteria in the rumen. Bacteria in the family Prevotellaceae play an important role in the excretion of nutrients such as cellulose and pectin and also play an important role in rumen digestion, as well as the ability to regulate lactic acid in the rumen, thereby improving the intrarumen environment (35). The dominant genera in this experiment are consistent with dominant studies with similar results (36).

In the current study, we predicted the function of the yak rumen microbial community using PICRUSt 2. The genes related to protein metabolism were enhanced in yaks in the F group. This suggests that the ruminal microflora of the F group produce large amounts of protein, which provide the host with raw materials such as protein to sustains life and normal metabolism. The increase in the percentage of concentrates increased the protein content of the rations, which created a more favourable environment for fermentation and the growth of cyto-lytic bacteria, which promote the participation of rumen microorganisms in the digestion and metabolism of nutrients. However, our results are based on predicted 16S rRNA only and may not be representative of the actual function of ruminal bacteria. Further macrogenomic analyses are needed to explore the mechanism of these genes functions in yaks fed high concentration diets. Network analyses can reveal positive and negative interactions between species (37). Negative interactions may weaken competitive relationships, while positive interactions may strengthen them back (38). In our study, these phenomena indicated that the competition between species in the G group was lower than that in the F group, and thus the rumen microecological community structure was more closely related.

### 4.3 Effects of natural grazing group and intensive feeding group on the rumen microbial fungi of yaks

Diet has been shown to significantly influence the population structure of rumen fungi in previous studies (39). Using high-throughput sequencing data to extract ITS genes, researchers have detected Ascomycetes and Ascomycetes in the rumen contents of cattle, suggesting that these fungi may represent populations of epiphytic fungi inhabiting pasture and other forages, rather than being directly involved in rumen fermentation. Other researchers have suggested that these fungi may play a role in digestive metabolism and may scavenge oxygen from the rumen, thereby maintaining a relatively anaerobic environment for other microorganisms. For instance, Atasoglu (22) reported a positive correlation between the growth of rumen fungi and the nutrient composition of the diet, with higher numbers of fungi observed under diets with high cellulose content. Our results are consistent with these previous findings (40), demonstrating that differences in fungal composition in the rumen were observed across different diets. For rumen fungi, research shows that rumen oomycetes have fiber-degrading enzyme activity, which can effectively degrade fiber and other substances in the diet and improve the utilization rate of coarse fiber (41). Orpinomyces can secrete plant cell wall degrading enzymes, which is conducive to the degradation and conversion of fiber materials (42).

## 5 Conclusion

In fact, intensive feeding altered the ruminal fermentation and composition of the microflora in yaks, which in turn affected their functions. Further improved efficient metabolism and contributed to yak growth. This study provides new knowledge about the rumen microbiome of yaks and may help to understand their adaptation to high-altitude environments. At the same time, it also provides theoretical guidance for natural grazing yaks in the plateau during the actual production.

## Data availability statement

The original 16S rRNA/ITS data were available in the NCBI SRA database with accession numbers PRJNA10366001 and PRJNA1034800s.

## Ethics statement

The animal study was reviewed and approved by the animal protection and utilization committee of Yunnan Agricultural University, China (protocol # 2017-0081), and there was compliance with the guidelines of the Laboratory Animal Ethics Committee in experimental animal handling. Written informed consent was obtained from the owners for the participation of their animals in this study.

## Author contributions

SH: Data curation, Methodology, Writing – original draft, Writing – review & editing, Conceptualization, Investigation, Visualization. ZY: Data curation, Investigation, Methodology, Writing – original draft. SD: Conceptualization, Data curation, Investigation, Methodology, Writing – review & editing. ZW: Conceptualization, Data curation, Investigation, Methodology, Writing – review & editing. SZ: Conceptualization, Data curation, Investigation, Methodology, Writing – review & editing. RW: Conceptualization, Methodology, Visualization, Writing – review & editing. QL: Methodology, Project administration, Supervision, Writing – review & editing. HM: Conceptualization, Funding acquisition, Methodology, Project administration, Writing – review & editing. DW: Conceptualization, Funding acquisition, Investigation, Methodology, Project administration, Visualization, Writing – review & editing.
